# Population-based study of high plasma C-reactive protein concentrations among the Inuit of Nunavik

**DOI:** 10.3402/ijch.v71i0.19066

**Published:** 2012-10-17

**Authors:** Marie-Eve Labonté, Eric Dewailly, Marie-Ludivine Chateau-Degat, Patrick Couture, Benoît Lamarche

**Affiliations:** 1 Institute of Nutraceuticals and Functional Foods, Laval University, Québec, QC, Canada; 2 Axe santé des populations et environnement, Centre de recherche du Centre Hospitalier Universitaire de Québec, Québec, QC, Canada; 3 Lipid Research Center, Centre Hospitalier Universitaire de Québec, Québec, QC, Canada

**Keywords:** Nunavik, risk factors, waist circumference, aging, sex, systolic blood pressure, Inuit, C-reactive protein, prevalence

## Abstract

**Background:**

The shift away from traditional lifestyle in the Inuit population over the past few decades has been associated with an increased prevalence of coronary heart disease (CHD) risk factors such as obesity, high blood pressure (BP) and diabetes. However, the impact of this transition on the pro-inflammatory marker high-sensitivity C-reactive protein (hs-CRP) has not been documented.

**Objectives:**

To examine the prevalence of elevated plasma hs-CRP concentrations in Inuit from Nunavik in the province of Quebec (Canada) and identify anthropometric, biochemical and lifestyle risk factors associated with elevated hs-CRP.

**Design:**

A population-representative sample of 801 Inuit residents from 14 villages of Nunavik, aged between 18 and 74 years, was included in the analyses. Subjects participated in a clinical session and completed questionnaires on lifestyle. Multivariate logistic regression was used to determine risk factors for elevated hs-CRP.

**Results:**

Elevated plasma hs-CRP concentrations (≥2 mg/L) were present in 32.7% (95% confidence interval (CI) 29.5–35.8) of the Inuit adult population and were more prevalent among women than among men (36.7% vs. 29.0%, p=0.007). Multivariate logistic regression analysis indicated that every 1 mmHg increase in systolic BP was associated with a 3% increase in the odds of having hs-CRP concentrations ≥2 mg/L in the Inuit population (95% CI 1.01–1.04). The combination of older age (≥50 vs. <30 years) and elevated waist circumference (gender-specific cut-off values) in a multivariate logistic model was also associated with a 13.3-fold increase in the odds of having plasma hs-CRP concentrations ≥2 mg/L (95% CI 5.8–30.9).

**Conclusions:**

These data indicate that elevated hs-CRP is relatively prevalent among Inuit with values that are similar to those seen in Canadian Caucasian populations. Sex, age, waist circumference and systolic BP are major factors that increase the risk of this inflammatory phenotype among Inuit from Nunavik, despite their different lifestyle background compared with Caucasians.

Inuit populations have experienced an important transition from a traditional to a modernized lifestyle over the past decades (1). This shift away from the traditional Inuit lifestyle has been associated with an increased prevalence of coronary heart disease (CHD) risk factors such as obesity (2,3), high blood pressure (BP) (2) and diabetes (4). High-sensitivity C-reactive protein (hs-CRP), a pro-inflammatory marker that has previously been shown to independently predict CHD outcomes in Caucasian populations (5,6), has recently been positively associated with cardiovascular disease (CVD) prevalence and carotid intima media thickness in the Inuit population of Alaska (7,8). Such data in Canadian Inuit populations have not been described yet.

This study investigated for the first time the prevalence of elevated plasma hs-CRP concentrations in Inuit from Nunavik in Northern Quebec and identified its risk factors, including anthropometric, biochemical and lifestyle characteristics, using unique data from the Nunavik Inuit Health Survey (NIHS) 2004 entitled “Qanuippitaa? – How are we?” (9).

## Methods

The present cross-sectional study included Inuit residents aged 18 years or more who participated in the extensive NIHS between 27 August and 1 October 2004. Residents of collective dwellings (i.e. hotels, hospitals and jails) and households in which there were no Inuit aged 18 years or more were excluded. The survey used a stratified random sampling of private Inuit households across the 14 coastal villages of Nunavik, Quebec. The assumption was that recruiting members of households rather than specific individuals would increase coverage of the target population. To obtain a good representation of each community, a proportional allocation of sample units based on households was chosen. Details of the sampling process and other methodological aspects are reported elsewhere (9).

Ethics committees of Laval University and Institut national de santé publique du Québec approved the survey. All participants provided written informed consent after watching a video describing the study.

### Clinical measures

The NIHS data collection included a 3-hour clinical session conducted by research team nurses aboard the Canadian Coast Guard ship (CCGS) Amundsen, which visited each of the 14 coastal villages of Nunavik. During the session, participants had to answer a clinical questionnaire. They also had a blood test, and physical measurements were taken.

Height, body weight, body fat composition and waist circumference measurements were described previously (10). Elevated waist circumference was defined as ≥90 cm in men and ≥80 cm in women according to the International Diabetes Federation (IDF) classification of central obesity for First Nations (11). BP was measured according to the Canadian Coalition for High BP technique (12). The presence of the metabolic syndrome (MetS) was determined using the IDF classification in First Nations (11). Diagnosis of type 2 diabetes status was based on self-report.

Participants were advised to fast for at least 8 hours prior to blood sample collection. Collected blood samples were centrifuged and stored at −80°C on-board the CCGS Amundsen and then sent at the Centre Hospitalier Universitaire de Québec for analyses of the cardio-metabolic risk factors (2). Plasma hs-CRP concentrations were measured using a commercially available, highly sensitive CRP assay (Behring Latex-Enhanced on the Behring Nephelometer BN-100; Behring Diagnostic, Westwood, Mass) and the calibrators provided by the manufacturer (N Rheumatology Standards SL; Behring Diagnostic). The mean inter-assay coefficient of variation for plasma hs-CRP concentrations was less than 1% at low and high plasma hs-CRP concentrations, as previously described (13).

### Lifestyle and socio-economic data

Participants completed an individual questionnaire administered in face-to-face interviews. It collected, among other information, data on living habits (e.g. smoking, physical activity) and socio-economic characteristics (e.g. education level). A confidential questionnaire was self-administered to participants to document more delicate subjects such as alcohol use. Examples of the questionnaires can be viewed in the methodological report of the survey (9). Classification of participants according to smoking status, drinking habits, physical activity levels and education level is described in Supplemental File 1.

### Statistical analyses

Elevated plasma hs-CRP concentrations were defined as ≥2.0 mg/L, as suggested by the 2009 Canadian guidelines for the diagnosis and treatment of dyslipidemia and prevention of CVD in the adult (14). As body mass index (BMI) may overestimate the prevalence of overweight and obesity among the Inuit (15), we used waist circumference and body fat composition (%) to evaluate the impact of adiposity on hs-CRP concentrations.

Statistical analyses were performed using the SAS software (version 9.2; SAS Institute, Cary, NC). Differences in characteristics of men versus women were assessed using Student's t-tests for continuous variables and the chi-square test for categorical data. The chi-square test was also performed to compare the prevalence of elevated plasma hs-CRP concentrations between subgroups of individuals stratified on the basis of sex (men/women), age (<30, 30–49, ≥50 years), waist circumference (low/high), and MetS (yes/no).

Multivariate logistic regression analysis was used to characterize the risk of elevated plasma hs-CRP concentrations according to lifestyle, anthropometric and biochemical variables. Odds ratio (OR) with 95% confidence intervals (CI) were calculated for each variable individually, using a multivariate model that included sex, age, waist circumference, and smoking as covariates. The model also took into account missing values for each of the predictor variables. To estimate missing data for continuous variables, multiple imputation (MI procedure in SAS) was performed prior to logistic regression analysis using the Markov Chain Monte Carlo method with a single chain to create 5 imputations (16). Missing data for categorical variables were adjusted for in the logistic model by including, for each variable, a separate group representing subjects with missing data. Because results from “missing data” subgroups are not interpretable and data assumed to be missing at random, their respective OR are not presented. Percent missing data for each independent variable in the models was 0% (no missing data) for sex and age; 0.4% for low-density lipoprotein cholesterol (LDL-C), high-density lipoprotein cholesterol (HDL-C), triacylglycerol (TG), and glucose; 0.9% for insulin; 3.1% for systolic and diastolic BP; 4.0% for waist circumference; 6.5% for smoking; 8.0% for physical activity; 8.2% for education level; and 23.5% for drinking habits. Finally, all variables were analysed simultaneously in a single multivariate logistic model to determine overall which risk factors remained significant and independent from one another. Two-way interactions among variables were also investigated using specific interaction terms in the multivariate logistic model. Significant interactions are presented graphically using different strata of relevant variables.

All data were analysed by the bootstrap technique in order to account for the complex sampling strategy employed and to correct for related sampling errors (SURVEY procedure in SAS or SUDAAN software (RTI International, NC) when the procedure was unavailable in SAS). All analyses were weighted to achieve population representativeness. Weights were adapted to the non-response rate of each measurement instrument. Since all results were weighted, the number of participants included in analyses is indicated for informational purposes only. Non-normally distributed variables were log-transformed prior to analysis. Statistical significance was set at a p-value of <0.05.

## Results

**Fig. 1:**
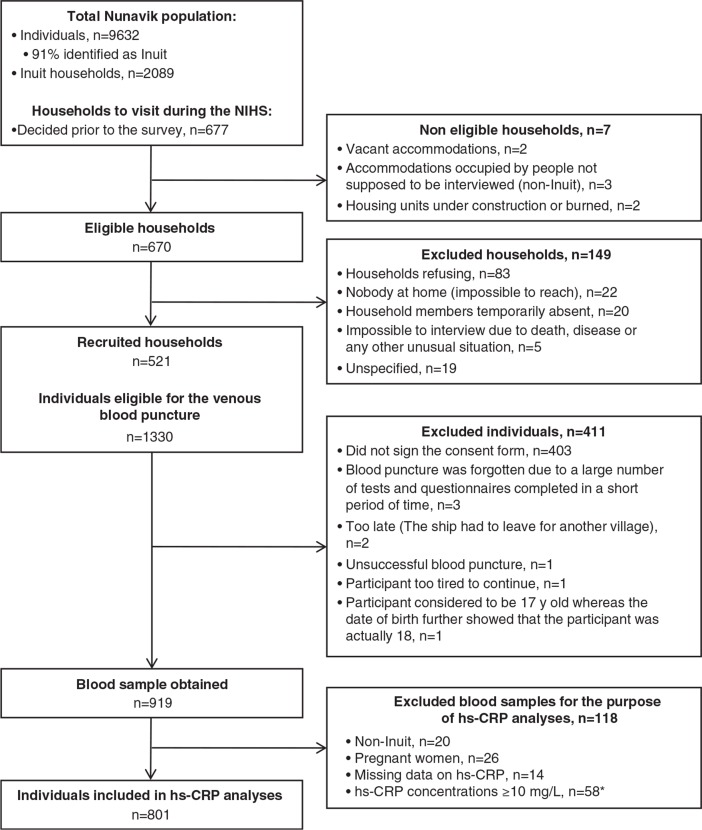
Flow of participants through the Nunavik Inuit Health Survey 2004. *58 subjects were excluded because they had hs-CRP concentrations ≥10 mg/L, which is indicative of an acute inflammatory response (17).

Figure 1 illustrates the flow of participants throughout the study. Analyses were based on a sample of 801 Inuit adults among 919 recruited participants from whom a blood sample was taken. Overall, 20 participants were excluded from the analyses because they were non-Inuit, 26 women were excluded because they were pregnant and 14 subjects were excluded because of missing data on hs-CRP concentrations. Also, 58 subjects (7.0% of the population, 95% CI 5.3–8.7) were excluded because their hs-CRP concentrations were ≥10 mg/L, which is indicative of an active acute inflammatory response (17).

Characteristics of the participants, stratified by sex, are shown in Table I. Compared with women, men were generally characterized by a deteriorated metabolic profile, including lower plasma HDL-C and apolipoprotein (apo) AI concentrations as well as higher total-C/HDL-C ratio and BP (all p<0.0001). Women had higher plasma interleukin (IL)-6 and tumour necrosis factor-alpha (TNF-α) concentrations than men (both p=0.006).

**Table I:** Characteristics of a random sample of the Nunavik Inuit adult population

Characteristics	Men^a^	Women^a^	p^b^
Age (year)^c^	35.9±0.37	36.7±0.33	0.09
Weight (kg)^c^	73.8±0.83	65.4±0.68	<0.0001
BMI (kg/m^2^)^c^	26.8±0.26	27.6±0.27	0.03
Body fat (%)^c^	20.9±0.38	31.6±0.41	<0.0001
Waist circumference (cm)^c^	90.8±0.66	91.3±0.64	0.62
Cholesterol (mmol/L)			
Total–C^c^	4.9±0.05	5.1±0.04	0.0007
LDL–C^c^	2.8±0.04	2.7±0.04	0.17
HDL–C^c^	1.5±0.02	1.8±0.02	<0.0001
Total-C/HDL-C ratio^c^^,^ d	3.5±0.06	3.0±0.04	<0.0001
Triacylglycerol (mmol/L)^c^^,^ d	1.2±0.04	1.2±0.03	0.12
Apolipoproteins			
ApoB100 (g/L)	0.9±0.01	0.9±0.01	0.35
ApoAI (g/L)	1.6±0.01	1.8±0.02	<0.0001
Blood pressure (mmHg)			
Systolic^c^	122.1±0.64	114.9±0.58	<0.0001
Diastolic^c^	75.5±0.49	72.5±0.39	<0.0001
Inflammatory markers			
hs-CRP (mg/L)^d^	1.8±0.09	2.0±0.10	0.38
IL-6 (pg/mL)^d^	2.1±0.09	2.3±0.09	0.006
TNF-α (pg/mL)^d^	2.1±0.10	2.5±0.13	0.006
Insulin (pmol/L)^c^^,^ d	62.7±3.15	67.1±2.61	0.004
Fasting glucose (mmol/L)^c^^,^ d	4.6±0.05	4.6±0.04	0.38
Physical activity (≥3.5 h/week, %)	52.7	39.3	0.0002
Smoking (current, %)	75.9	81.4	0.03
Drinking (≥1 drink/day, %)	30.1	23.2	0.03
Education level (≥ high school, %)	23.3	22.6	0.80

Apo = apolipoprotein; BMI = body mass index; C = cholesterol; HDL = high-density lipoprotein; hs-CRP = high-sensitivity C-reactive protein; IL-6 = interleukin-6; LDL = low density lipoprotein; TNF-α = tumour necrosis factor-α.

a n = 280–367 for men and n = 333–434 for women, depending on the variable. Values are means±SEM unless stated otherwise. All means were weighted to achieve population representativeness. Hence, the number of participants is indicated for informational purposes only.

b p-Values based on a Student's t-test except for physical activity, smoking, drinking and education level which were determined using the Chi-square test in SAS.

c These data have already been reported in a previous publication (3).

d Variables were log transformed prior to analysis, but non transformed data are presented for better interpretability.

**Fig. 2:**
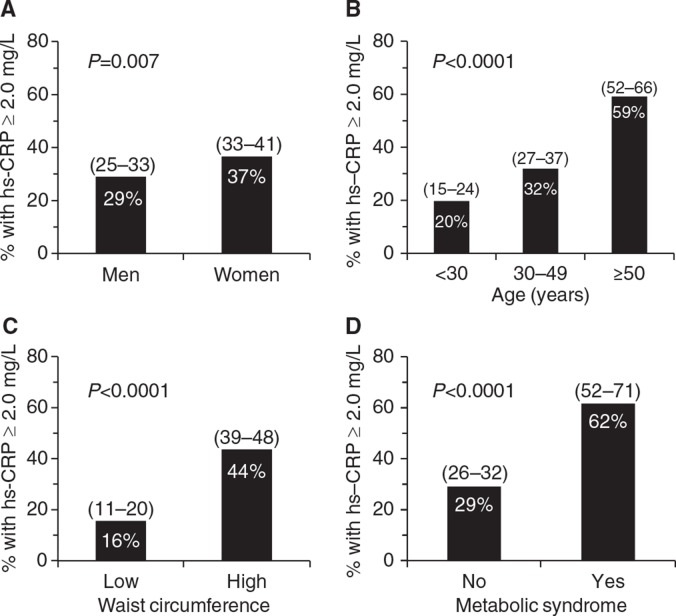
Population prevalence of elevated hs-CRP concentrations among Inuit from Nunavik. Note: hs-CRP = high-sensitivity C-reactive protein; MetS = metabolic syndrome. Population prevalence of elevated hs-CRP concentrations (≥2.0 mg/L) is presented according to sex (A), age (B), waist circumference (C) and the presence of MetS (D). p-Values for between-groups differences in frequencies were obtained using the Chi-square test. Values within parentheses are 95% confidence intervals. Prevalence values are unadjusted for other variables. In panels C and D, high waist circumference cut-offs (≥90 cm in men and ≥80 cm in women) and MetS criteria are those suggested by the International Diabetes Federation (11).

Estimated proportion of individuals with plasma hs-CRP concentrations ≥2.0 mg/L among the Nunavik Inuit population was 32.7% (95% CI 29.5–35.8). The estimated proportion of the population with plasma hs-CRP concentrations >3.0 mg/L was 21.4% (95% CI 18.7–24.0). As shown in Fig. 2, elevated plasma hs-CRP concentrations (≥2.0 mg/L) were more prevalent among women than among men (p=0.007). The prevalence of elevated hs-CRP concentrations also increased with age, waist circumference and presence of MetS (Fig. 2).

As shown in Table II, age and waist circumference were significantly associated with the odds of having plasma hs-CRP concentrations ≥2.0 mg/L (all p≤0.003) in a multivariate logistic model that also included sex and smoking as covariates. HDL-C, TG (log transformed), insulin (log transformed), systolic BP and diastolic BP were also significantly associated with the odds of having plasma hs-CRP concentrations ≥2.0 mg/L (all p≤0.008; Table II) when considered individually with adjustment for sex, age, waist circumference and smoking. When considering all risk factors simultaneously in a single multivariate model, only age, waist circumference and systolic BP remained significantly and independently associated with the odds of having plasma hs-CRP concentrations ≥2.0 mg/L (all p≤0.02; not shown). Female sex was also significantly and independently associated with the odds of having plasma hs-CRP concentrations ≥2.0 mg/L in this single multivariate model (OR=1.54; 95% CI 1.04–2.28; p=0.03; not shown). Replacing waist circumference with percent body fat in the various multivariate logistic models had no impact on results (not shown). Further adjustment for the use of antihypertensive, diabetic and lipid-lowering medications or excluding subjects on these medications (7.5%, 3.2% and 5.1% of participants, respectively) also yielded similar results (not shown).

**Table II:** Adjusted odds ratio (OR) for elevated high-sensitivity C-reactive protein (hs-CRP) concentrations according to demographic, anthropometric, biochemical and lifestyle risk factors

Variables	Adjusted OR hs-CRP ≥2.0 mg/L^a^	95% CI	p
Sex			
Men	1.00		
Women	1.06	0.78–1.44	0.71
Age (years)			
<30	1.00		
30–49	1.63	1.18–2.27	0.003
≥50	4.93	3.27–7.42	<0.0001
Waist circumference (cm)^b^			
Low	1.00		
High	3.48	2.19–5.53	<0.0001
LDL-C (mmol/L)	0.99	0.82–1.20	0.92
HDL-C (mmol/L)	0.51	0.32–0.81	0.005
Log TG (mmol/L)	3.38	1.59–7.19	0.002
Log fasting glucose (mmol/L)	3.15	0.42–23.61	0.26
Log insulin (pmol/L)	2.20	1.24–3.90	0.007
Systolic BP (mmHg)	1.03	1.01–1.04	0.0001
Diastolic BP (mmHg)	1.02	1.01–1.04	0.008
Physical activity^c^			
<3.5 h/week	1.00		
≥3.5 h/week	0.83	0.61–1.13	0.23
Smoking status^d^			
Non-smokers	1.00		
Ex-smokers	0.51	0.25–1.03	0.06
Current smokers	0.61	0.34–1.10	0.10
Drinking habits^e^			
Never	1.00		
Light	0.66	0.39–1.11	0.12
Moderate	0.73	0.40–1.32	0.30
Heavy	0.80	0.41–1.59	0.53
Education level^f^			
<High school	1.00		
=High school	1.05	0.68–1.64	0.82
>High school	1.25	0.69–2.28	0.46

BP=blood pressure; C=cholesterol; CI=confidence intervals; HDL=high-density lipoprotein; hs-CRP=high-sensitivity C-reactive protein; LDL=low density lipoprotein; OR=odds ratio; TG=triacylglycerol.

a OR and 95% CI were determined for each variable individually, using a multivariate logistic regression model in SAS that included sex, age, waist circumference and smoking status as covariates. The model also took into account missing values for each of the predictor variables.

b High waist circumference cut-offs were ≥90 cm in men and ≥80 cm in women.

c Physical activity < or ≥3.5 h/week for at least 1 of the 4 seasons of the previous year.

d “Non-smokers” = Inuit not smoking at the time of interview, who also never smoked up to 100 cigarettes in their lifetime; “Ex smokers” = Inuit not smoking at the time of interview, who smoked a total of 100 cigarettes or more in their lifetime; “Current smokers” = occasional and daily smokers.

e “Never drinkers”=no alcohol consumed during the previous year; “Light drinkers”=<1 drink/day; “Moderate drinkers”=1–2 drinks/day; “Heavy drinkers”=>2 drinks/day.

f The “=high school” category represents Inuit who completed high school as well as those who undertook a *partial* training in a community college, a trade school, a private commercial college, a technical institute, a CEGEP or a nursing school.

**Fig. 3:**
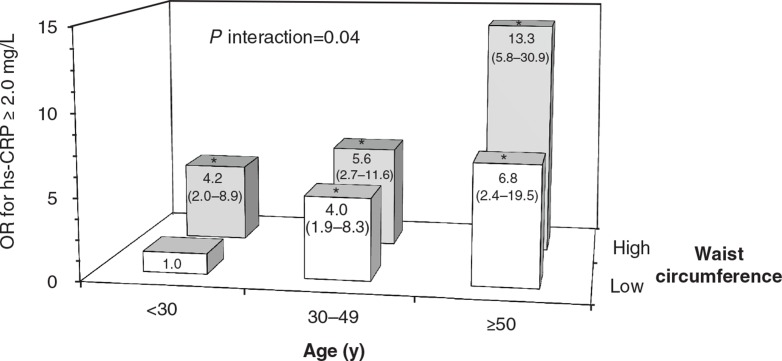
Odds ratio (OR) for elevated high-sensitivity C-reactive protein (hs-CRP) concentrations according to the combined impact of age and waist circumference. Note: A significant age*waist circumference multiplicative interaction (p interaction = 0.04) was found on the odds of having elevated hs-CRP concentrations (≥2.0 mg/L) among the Nunavik Inuit population, using a multivariate logistic regression model. To illustrate the interaction, 6 groups were created based on the combination of different strata of age (<30, 30–49, ≥50 years) and waist circumference (low/high) and were simultaneously entered into a logistic model, with the combination of age <30 years and low waist circumference as the reference group (OR = 1). OR and 95% confidence intervals (in parentheses) were obtained for each group and are adjusted for high-density lipoprotein cholesterol, triacylglycerol (log transformed), insulin (log transformed), systolic blood pressure (BP), diastolic BP, sex and smoking. Positive associations of female sex and systolic BP with elevated hs-CRP concentrations remained significant in this model (p ≤ 0.01). High waist circumference cut-offs were ≥90 cm in men and ≥80 cm in women, as suggested by the International Diabetes Federation (11). *OR significantly higher than the reference group, p ≤ 0.0003.

A 2-way multiplicative interaction was observed between age and waist circumference on the odds of having elevated plasma hs-CRP concentrations (p interaction=0.04) in a multivariate logistic model that adjusted for HDL-C, TG (log transformed), insulin (log transformed), systolic BP, diastolic BP, sex and smoking. This interaction is presented graphically in Fig. 3 using combinations of different strata of age (<30, 30–49, ≥50 years) and waist circumference (low/high based on sex-specific cut-points). The combination of older age (≥50 vs. <30 years) and abdominal obesity (≥90 cm in men and ≥80 cm in women vs. normal waist) was associated with a 13.3-fold increase in the odds of having high hs-CRP concentrations (95% CI 5.8–30.9, p<0.0001) independent of other risk factors associated with high hs-CRP concentrations.

## Discussion

To the best of our knowledge, this population-based study was the first to determine the prevalence of elevated plasma hs-CRP concentrations, defined as ≥2.0 mg/L, in the Inuit population from Nunavik. Our results showed that elevated hs-CRP is present in nearly one-third of the Inuit adult population. We identified sex, age, waist circumference and systolic BP as the primary risk factors of having plasma hs-CRP concentrations ≥2.0 mg/L in this population. In addition to their independent association with elevated hs-CRP concentrations, age and waist circumference appear to exert a synergistic influence on hs-CRP concentrations.

Previous studies have shown that the prevalence of high hs-CRP concentrations in Canadian aboriginal populations is higher than among individuals of European ancestry (18,19). Anand et al. (19) have indeed shown that 54.8% of Aboriginals from the Six Nations Reservation in Ontario, Canada, had elevated hs-CRP concentrations based on a cut-off of 3.0 mg/L compared with 25.0% of Canadians of European origin. Only 21.4% of the Nunavik Inuit population in the present study had hs-CRP concentrations >3.0 mg/L. The prevalence of elevated hs-CRP concentrations among Inuit adults thus appears to be less than half than in other Canadian aboriginal populations. The prevalence in the Inuit population is actually comparable to the prevalence observed in Caucasians, despite the fact that other CHD risk factors such as obesity and smoking are more prevalent in Inuit versus Caucasians (10). Our observation is population-based and therefore most likely to represent the true prevalence of elevated hs-CRP in the Nunavik Inuit population. Further investigations are warranted to determine if this relatively low prevalence of high hs-CRP is also seen in other circumpolar populations.

Our data derived from multivariate logistic regression analysis are consistent with previous studies that have identified female sex (18–20), aging (21,22) and obesity indices (23,24) as the most significant and independent predictors of high hs-CRP concentrations. Despite a traditional lifestyle background, the Inuit population appears to share similar risk factors for elevated hs-CRP concentrations as Caucasian populations. This observation is further supported by similar results gathered in the Canadian Oji-Cree population (18) and in urban Chinese men facing a rapid lifestyle transition (25).

Very few observational studies have reported an association between systolic BP and elevated plasma hs-CRP concentrations. Consistent with our data in Inuit, a study in Australian men and women showed that hypertension (vs. no hypertension) predicted an increased risk of having elevated hs-CRP concentrations (OR=1.6 in men and 1.4 in women for hs-CRP concentrations >3.0 mg/L) even after adjustment for BMI, diabetes status, total-C, HDL-C, TG, smoking, exercise and, in women, menopausal status and hormone replacement therapy (26). The positive association between systolic BP and hs-CRP may reflect the activation of the renin-angiotensin system (RAS) (27,28). Indeed, one of the main products of the RAS is angiotensin II (Ang II), a powerful vasoconstrictor that has been shown to induce inflammation in the vascular wall (28) as well as in the adipose tissue (29). Increasing evidence suggests that Ang II stimulates the secretion of pro-inflammatory cytokines, such as TNF-α, IL-6, or IL-1β, from vascular smooth muscle cells (30,31), immune cells (32) and adipocytes (33) through activation of the transcription factor nuclear factor kappa B (NF-κB) (29,34). IL-6 has been shown to up-regulate the messenger RNA (mRNA) transcription of CRP in hepatocytes while IL-1β stimulates CRP mRNA translation (35).

Finally, the combination of different strata of age and waist circumference in a multivariate model showed that Inuit of older age with an elevated waist circumference had a 13-fold increase in their odds of having elevated plasma hs-CRP. Although highly significant, this estimate has a relatively high degree of imprecision, and additional studies are therefore needed to confirm that Inuit people portrayed by these characteristics should be targeted in public health interventions aimed at reducing the risk for elevated hs-CRP in the Inuit population. Most importantly, further research is warranted to investigate how elevated hs-CRP concentrations relate to future CHD events especially in Inuit showing these attributes.

### Limitations

First, the cross-sectional design of the study precludes identification of causal associations between elevated hs-CRP concentrations and identified risk factors. However, the main correlates of hs-CRP have been determined using a large and representative sample of the Nunavik Inuit population as well as standardized methods for collection of risk factor data. Although our analyses were adjusted for several confounders, other factors such as infectious agents (e.g. *Helicobacter pylori*, zoonoses) may have affected hs-CRP concentrations upward in the Inuit population. However, the association observed between inflammation and the MetS in the Persian Gulf Healthy Heart Study was independent of pathogen burden (36). There is also recent evidence indicating that obesity status remains a strong predictor of elevated hs-CRP concentrations even above 10 mg/L (37).

## Conclusion

Elevated hs-CRP concentrations are relatively prevalent among the Nunavik Inuit adult population with values that resemble those seen in Canadian Caucasian populations, but that are significantly lower than in other native populations. Well-known risk factors for elevated hs-CRP, including female sex, aging and waist circumference, were identified as major correlates of this inflammatory phenotype among Inuit, despite their different lifestyle background compared with Caucasians. The extent to which elevated hs-CRP concentrations predict future CHD events in the Nunavik Inuit population requires further assessment using longitudinal, prospective data.
